# Reducing the Complexity of an Agent-Based Local Heroin Market Model

**DOI:** 10.1371/journal.pone.0102263

**Published:** 2014-07-15

**Authors:** Daniel Heard, Georgiy V. Bobashev, Robert J. Morris

**Affiliations:** 1 Department of Statistical Science, Duke University, Durham, North Carolina, United States of America; 2 RTI International, Research Triangle Park, North Carolina, United States of America; University Toulouse 1 Capitole, France

## Abstract

This project explores techniques for reducing the complexity of an agent-based model (ABM). The analysis involved a model developed from the ethnographic research of Dr. Lee Hoffer in the Larimer area heroin market, which involved drug users, drug sellers, homeless individuals and police. The authors used statistical techniques to create a reduced version of the original model which maintained simulation fidelity while reducing computational complexity. This involved identifying key summary quantities of individual customer behavior as well as overall market activity and replacing some agents with probability distributions and regressions. The model was then extended to allow external market interventions in the form of police busts. Extensions of this research perspective, as well as its strengths and limitations, are discussed.

## Introduction

We present an example of agent-based model reduction, which is defined as simplifying a detailed agent-based model while preserving the model's statistical characteristics. In this sense, the reduced model acts as an emulator of the original, full model. A more traditional model-building approach proceeds in the reverse order: starting as simple as possible and adding more details only if the model fails to sufficiently reproduce reality. This approach, which can be described as a top-down process, relies on a solid understanding of causal mechanisms. Because many locally different causal mechanisms can produce the same aggregate behavior, a simple model based on the wrong causal mechanisms could suggest erroneous system response to real intervention [1]. A model built using the alternative bottom-up process starts with a detailed description of the observed behaviors with the goal of providing higher fidelity to the causal mechanisms. However, because it is not always clear which behavioral details are critical for reproducing the observed global patterns, the level of detail in such models may go beyond what is necessary to obtain the relevant information from the simulation. This approach has been discussed before in the context of infectious disease modeling [23] and in the context of social segregation [24]. However in these studies the focus was primarily on the concept of model reduction, not on the identification of the driving mechanisms. In this paper we perform a bottom-up model reduction process on a model that simulates a local heroin market and that has been published elsewhere [2], [3].

Despite significant funding to fight heroin use, heroin markets show surprising resilience as described in recent news articles and reports [4]–[6]. This resilience suggests that better understanding of the actual functioning of drug markets is needed. At the same time, a number of economic models have been developed to identify and forecast market components and operations [7]–[10]. Ethnographic methods applied to medical and cultural anthropology show that heroin markets do not follow traditional economic patterns. As described in Hoffer [11], “The difference here is subtle but clear: dealing heroin is a social behavior with economic outcomes rather than an economic behavior with social outcomes.” On the local market level, the economics of heroin transactions involve customers purchasing the drug directly from street dealers or private dealers, or through street brokers serving as intermediaries.

A recently developed agent-based model called the illicit drug market simulation (IDMS) used ethnographic data [2] to describe in detail the functioning of the Larimer open-air heroin market in Denver, Colorado, from 1992 to 1996 [2], [3]. This paper focuses on methods for developing a reduced version of the IDMS to increase efficiency while maintaining fidelity to the full model. We also examine the utility of a reduced model and the methodology for developing the reduced model. Section 2 presents an overview of the IDMS model. Section 3 describes the procedure for model reduction. Sections 4 and 5 present comparisons of the full and reduced model. Finally, section 6 presents a discussion and concluding remarks.

## Model Overview

The IDMS contains six types of agents: customers, street dealers, street brokers, private dealers, police, and homeless.

Customer agents purchase and use heroin, and have addictions that grow and diminish according to pharmacodynamic characteristics. Customers seek to purchase a target amount that most closely matches their addiction level. Their income is replenished weekly, every 2 weeks, or monthly, and with random increases in money. Customers keep track of sellers from whom they purchase drugs and seek specific locations to purchase based on previous successful transactions.

Brokers act as intermediaries, transporting money from customer to dealer and returning the drug to the customer. Brokers take a portion of the heroin from transactions as a “tax” and use it themselves. Brokers are also the only agents who can introduce a customer to a private dealer.

Street dealers have a given inventory of heroin to sell. They sell the heroin for a fixed price and stop selling when either they have sold their entire inventory or their shift ends, whichever comes first. Sellers can be arrested and removed from the market for a period of time determined by the amount of heroin they possess at the time of arrest.

Private dealers sell inventories of heroin for a fixed price, similar to street dealers; however, private dealers operate outside of the open-air market. Private dealers only sell to customers or brokers they know.

Police patrol the market, randomly inspecting individuals and arresting any inspected individual found to possess heroin.

Homeless agents have no active role in the market and only serve to provide noise in the market through increased population.

All agents interact in regions designated as either public (representing the open-air market) or private (representing customer and private dealer residences).

In the simulations for the model, the initial agent counts were as follows: 200 customers, 20 street dealers, 25 street brokers, 25 private dealers, 100 homeless, and 1 police officer.

## Model Reduction

Our model reduction strategy was to approximate the complex decision processes of agents using statistical distributions and regression models, a method that has been explored for other complex agent-based models [12]. At each time step, agents determine their behavior based on their current state and the state of agents around them. As the number of agents increases, these processes can become quite demanding. The most complex component of the IDMS describes agent behavior in the open-air market, a publicly accessible, geographically structured area where the agents move around and interact with each other. Examples of interactions include customers meeting street dealers to purchase heroin and police making arrests. Because the market activity is driven by the behavior of the customer agents, our model reduction focused on simplifying the processes for determining customer interactions with other agents.

We began by examining the ethnographic material in Hoffer [11] and the function of the full model describing customer behavior in the heroin market. This behavior is driven by a set of rules under which a customer first decides to travel to the open-air heroin market or contact a private dealer in the case that one is known. If the decision is to go to the market, then, upon arriving at the market, the customer moves towards the likely location of a seller (dealer or broker) based on previous successful transactions. If the seller is not found, the customer moves around the market (via a random walk) trying to find one. After a customer makes a deal either with the dealer or a broker for a certain amount of heroin, the success of the transaction will depend on a number of factors. A deal could be unsuccessful as the result of a broker getting arrested on the way to deliver heroin or from an insufficient amount of heroin available. The amount of heroin available depends on the number of the dealers, the initial amount each dealer has when they arrive at the market, the number of customers and their demands. Because in the simplified model we are interested in the amount of heroin customers obtain and the amount of money spent in the market, based on our examination of market dynamics, we identify five outcomes to capture the customer behaviors of interest:

- The probability of obtaining heroin
- The amount of time spent in the market
- The probability of being arrested
- The probability of purchasing via a street broker (versus purchasing from a street dealer)
- The probability of receiving an invitation to purchase directly from a private dealer in the future

We selected these five outcomes because of their important effects on the dynamics of the market and the overall simulation. Although there are other outcomes that contribute to the simulation, these are among the most influential to the customer's addiction cycle. The probability of obtaining heroin is a critical factor for market efficiency. Higher probabilities lead to increased individual drug habits, which in turn lead to an increase in the amount of drug purchased. The probability of being arrested controls the number of customers in the market. The amount of time spent in the market is indicative of market efficiency. The probability of purchasing via a street broker and the probability of receiving an invitation to purchase from a private dealer are both related to the customer's ability to find alternative sellers of heroin. This is particularly important during periods of increased police activity when fewer street dealers are available.

In our reduced model, we removed all explicit customer interactions in the market and instead computed statistical approximations of the above five outcomes. An implication of this is that we eliminated the need for the function of police, street broker, street dealer, and homeless agents, all of whom had no active role outside the market. From the perspective of a customer's drug use and experience in the drug market, these other agents were necessary only inasmuch as they affected the customer's outcomes. By using a probabilistic approach to determine a customer's experience in the market, the individual behaviors of other types of agents were no longer consequential. The procedure causing heroin users to seek drugs as well as the pharodynamics of each heroin user in the reduced model, including their changing addiction levels and physical drug concentration levels, were kept the same as the full model. We kept these elements unchanged because they are not directly related to the complex decision processes that take place when customers are in the market, which was the main focus of our reduction. Additionally, this allowed us to maintain consistency across the full and reduced models and limit the set of model behaviors being examined.

Some of the market trip outcomes are conditional on others. In the full model, these relationships occur naturally because of the customer's agent-level interactions. When replacing the customer interactions with statistical approximations, we had to establish a decision hierarchy that reflected the conditional relationships between the outcomes. The decision hierarchy proceeds in the following way:

1. Compute the probability of obtaining the drug, p_o_. Using a Bernoulli trial with probability p_o_, determine the customer's market trip outcome, π_o_ ∈ {0,1}, where 0 represents failure and 1 represents success.
2. Conditional on π_o_, compute the time spent in the market, t_m0_ or t_m1_.
3. Conditional on π_o_ = 1, compute the probability of being arrested, p_a_. (Note that if π_o_ = 0, then p_a_ = 0.) Using a Bernoulli trial with probability p_a_, determine whether the customer was arrested.
4. Conditional on π_o_ = 1, compute the probability of purchasing via a street broker, p_sb_. Using a Bernoulli trial with probability p_sb_, determine the customer's purchase mode, π_sb_ ∈ {0,1}, where 0 represents a street dealer and 1 represents a street broker.
5. Conditional on π_sb_ = 1, compute the probability of being invited to purchase directly from a private dealer, p_i_. Using a Bernoulli trial with probability p_i_, determine whether the customer was invited to purchase directly from a private dealer.

### Time Step Analysis

Before starting the model reduction procedure, we first examined how changing the time step (the amount of time simulated by one “tick” of the model) would affect the summary statistics for our five key market trip outcomes. We sought the largest time step that would produce stable values of these summary statistics in the full model. Increasing the model's time step increases the run-time efficiency of the model by reducing the number of times agents must go through their decision processes during the 1-year simulation. However, because many of the agents have highly time-dependent behaviors, adjusting the time step could also cause important interactions not to occur.

**Figure 1:**
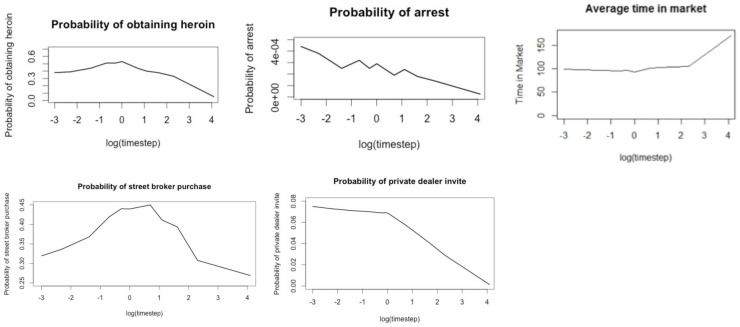
Plots of average summary statistics versus log time step, where zero corresponds to 1 The curves all show clear shifts as the time step moves away from 1

The time step used in the original IDMS model was 1 minute. We ran the model using the following values for the time step: 3, 6, 15, and 30 seconds; 1, 2, 3, 5, and 10 minutes; and 1 hour. Figure 1 demonstrates a clear trend of our summary statistics changing as the time step varies from 3 seconds to 1 hour. In particular, the probability of obtaining heroin, the probability of being arrested, and the probability of being invited to make private deals decrease as the time step increases beyond 1 minute. A possible explanation for this is the number of nonlinear relationships in the model, which could lead to the accumulation of linearization error. For example, for a customer to obtain heroin or to be arrested, he or she must be within the vicinity of a seller or police officer, respectively, at the end of a model tick. Because increasing the time step decreases the number of model ticks, larger time steps produce fewer opportunities for agents to end up within the vicinity of other agents. We also observed variation in the summary statistics for time steps smaller than 1 minute, although not as much as when increasing the time step beyond 1 minute. The probability of purchasing from a street broker and of being arrested show the most variation for smaller time steps.

Even at 2 minutes (0.69 on the log scale), most of the summary statistics depart from their values under the original time step. With the exception of probability of arrest, the summary statistics remain stable near their 1-minute values as the time step decreases. Based on these trends, we chose to maintain the original 1-minute time step for the model.

### Statistical Approximation of Market Trip Outcomes

To approximate the five key market trip outcomes, we considered two approaches. We began with the simpler of these two approaches and evaluated how well the results of the reduced model matched those of the full model. If the results were not sufficiently similar, we moved on to the more complicated approach. In the simpler approach, we produced the observed distribution of each outcome over the population of customer agents, and then we fit a statistical distribution to the observed distribution. We simulated the market trip outcome in the reduced model by taking a random draw from the statistical distribution. In the more complicated approach, we accounted for the heterogeneity in customer behavior by fitting a regression model to the observed data. We simulated the market trip outcome in the reduced model by using the regression model to predict the outcome. We looked to balance fidelity to the full model with parsimony and a limited number of variables when developing the regression models.

Given the five market outcomes we identified for analysis in model reduction, we began with the simplest approach of using distributions for the five outcomes. When we found simulation results not sufficiently similar to the full model, we elected to institute regressions for the outcomes based on the specific discrepancy between the full and reduced models. This required comparison not only of aggregate customer behavior, but also comparison of the trajectories of specific customer quantities in the simulations. In these instances, we used the trellis graphics as a starting point for the selection of independent variables for the regression models. This allowed us to examine how several variables were related to the market outcomes we identified, as it was not immediately clear which variable(s) would have adequate explanatory power as regression covariates. The trellis graphics plotted each of the five market outcomes against quantities such as the number of customers in the market, the number of dealers/brokers in the market, the amount of heroin sold, time of day, among others.

We ran 100 simulations of the full model to obtain estimates and distributions for the five key market trip outcomes. Because the model starts in a high-variation transient state before nearing a steady state by day 180, and because we were concerned with the model's behavior in its steady state, we used the model results starting at day 180 when computing our estimates and distributions.

We discuss the statistical approximation for each market trip outcome below.

### Probability of Obtaining Heroin

**Figure 2:**
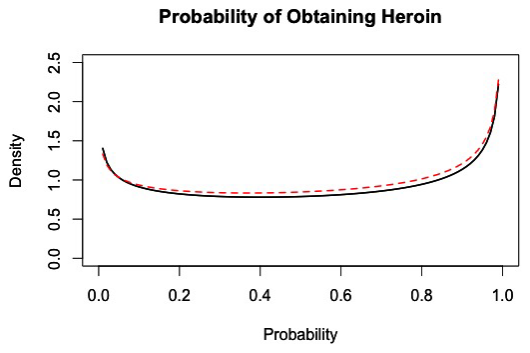
Distribution of the probability of obtaining heroin. The black line represents the density of success probability across all customers within each 1-hour interval of market activity, based on 100 simulations of the market from days 180 to 365. The dashed red line represents a fitted distribution, which is a Beta (0.835,0.718) distribution.

The overall average probability of obtaining heroin in the market is p_o_ = 0.53, with the distribution shown in Figure 2.

**Figure 3:**
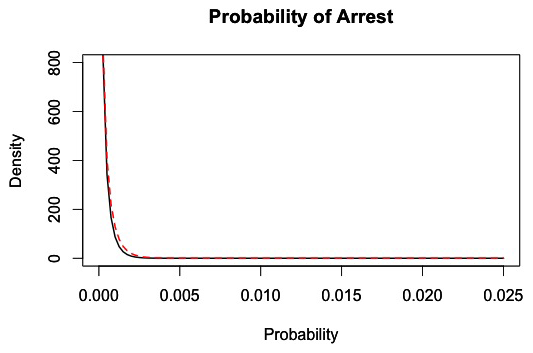
Distribution of the probability of being arrested. The black line represents the density of arrest probability across all customers within each 1-hour interval of market activity based on 100 simulations of the market from days 180 to 365. The dashed red line represents a fitted distribution, which is a Beta (0.43,1500) distribution.

The reduced model failed to produce results sufficiently similar to the full model when using this distribution to predict whether customers successfully obtain heroin. Although using a distribution produced the same average rate of customer success in market trips as the full model, this approach resulted in patterns of successes and failures that were not consistent with results from the full model. Therefore, we also fit a regression model to the data. Because obtaining heroin is the central component to customers' addiction cycle, this was the first outcome for which we implemented a regression. We constructed a mixed-effects model where data from each customer are represented by a random effect and within-customer responses are correlated. We considered the model's logic behind purchasing the drug and examined a number of independent variables that may contribute to the prediction. We found the strongest predictor to be the amount of heroin sold by street dealers during the day up to the time of the customer's attempted purchase, i.e. the more heroin has already been sold before the customer has arrived, the less is left, and thus is less likely to be found. Although the amount of heroin sold was the only variable among those we investigated which showed significant correlation with the probability of obtaining heroin, we investigated including additional predictors in the model, such as the amount of heroin the customer sought to purchase or number of sellers or customers present in the market, but none added to the quality of model fit. We thus obtained the following mixed-effects logistic regression model:
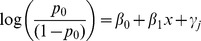
where x is the number of units of heroin sold by street dealers, the intercept is β_0_ = 10.19 with a standard error of 0.01588, and the coefficient for the units sold is β_1_ = −0.00115. The customer-level random effect γ_j_ has a variance of 0.68.

### Time Spent in the Market

The overall average amount of time a customer spends in the market is t_m_ = 93.8 minutes, but the distribution is bimodal. Closer examination of the modalities revealed that they are related to whether the user is successful in purchasing heroin, and so we decided to estimate these conditional distributions separately.

**Figure 4:**
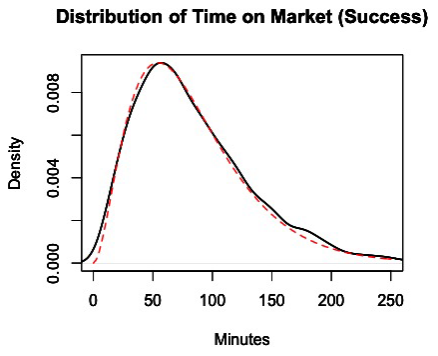
Distribution of time in the market, conditional on successfully obtaining heroin. The black line represents the density of the amount of time each customer spent in the market for individual successful market trips based on 100 simulations of the market from days 180 to 365. The dashed red line represents a fitted distribution, which is a Gamma (2.9,0.035) distribution.

Conditional on a customer successfully obtaining heroin, the average time in the market is t_m1_ = 78.3 minutes, with the distribution shown in Figure 4.

**Figure 5:**
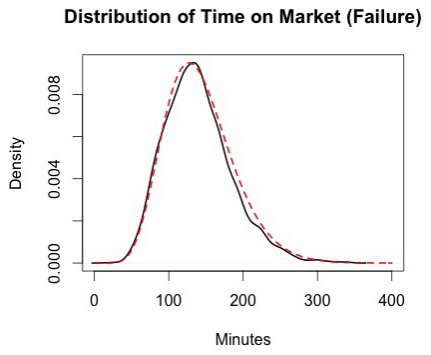
Distribution of time in the market, conditional on failure to obtain heroin. The black line represents the density of the amount of time each customer spent in the market for individual failed market trips based on 100 simulations of the market from days 180 to 365. The dashed red line represents a fitted distribution, which is a Gamma (9.11,0.072) distribution.

Conditional on a customer failing to obtain heroin, the average time in the market is t_m0_ = 126.5 minutes, with the distribution shown in Figure 5.

The reduced model produced acceptable results when using these distributions to predict the amount of time customers spend in the market. Thus, using regression models to improve the results was unnecessary in this case.

### Probability of Being Arrested

The overall average probability of a customer being arrested in the market is p_a_ = 0.00029, with the distribution shown in Figure 3.

The reduced model produced acceptable results when using this distribution to predict customer arrest. Thus, using a regression model to improve the results was unnecessary in this case.

### Probability of Purchasing via a Street Broker

**Figure 6:**
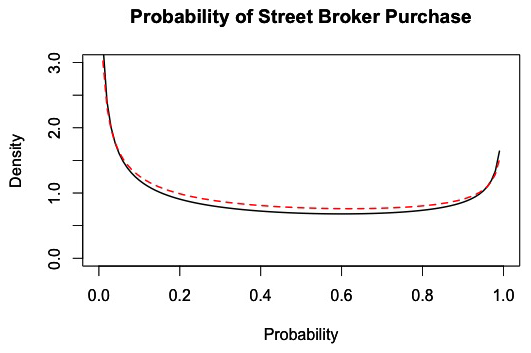
Distribution of the probability of purchasing via a street broker, conditional on successfully obtaining heroin in the market. The black line represents the density of the probability of a street broker purchase across all successful customer market trips within each 1-hour interval of market activity based on 100 simulations of the market from days 180 to 365. The dashed red line represents a fitted distribution, which is a Beta (0.61,0.76) distribution.

Conditional on a customer successfully purchasing heroin in the market, the overall probability of purchasing via a street broker is p_sb_ = 0.446, with the distribution shown in Figure 6. We enforced the condition of purchasing in the market to exclude all private dealer transactions when calculating the probability. Like the full model, customers in the reduced model choose to travel either to the public market or to a private dealer to obtain drugs. Because the intent of the reduced model was to simplify customer behavior in the public market only, we did not consider private dealer purchases in the reduction process.

The reduced model produced acceptable results when using this distribution to predict whether a customer purchases from a street broker. Thus, using a regression model to improve the results was unnecessary in this case.

### Probability of Receiving an Invitation to Purchase Directly From a Private Dealer in the Future

**Figure 7:**
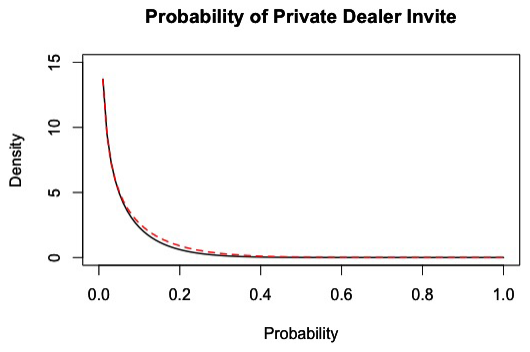
Distribution of the probability of being invited to purchase from a private dealer, conditional on successfully purchasing heroin via a street broker. The black line represents the density of the invite probability across all customers within each 1-hour interval of market activity based on 100 simulations of the market from days 180 to 365. The dashed red line represents a fitted distribution, which is a Beta (0.55,7.45) distribution.

Conditional on successfully purchasing heroin via a street broker, the overall probability of a customer being invited to purchase from a private dealer is p_i_ = 0.069, with the distribution shown in Figure 7.

The reduced model failed to produce results sufficiently similar to the full model when using this distribution to predict whether customers are invited to deal directly with a private dealer. Although the distribution approach produced similar relative proportions of customer purchases from private dealers, the trajectory of the number of customer purchases from private dealers was not consistent with results from the full model. Therefore, we also fit a regression model to the data. Based on examination of trellis graphics, we identified the strongest predictor to be the customer's number of successful heroin purchases via a street broker. None of the other quantities showed correlation with customer invitations to a private dealer and the inclusion of other covariates did not improve model fit. Thus, we constructed the following logistic regression model:
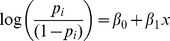
where x is the number of times the customer has purchased heroin via a street broker prior to the current attempted purchase, the intercept is β_0_ = −4.0763 with a standard error of 0.00762, and the coefficient for the number of successful street broker purchases is β_1_ = 0.00141.

## Model Comparison Results

The primary goal of our model reduction was to make the model simpler and more efficient while minimizing the impact on the model results. We made the model simpler and more efficient by replacing the complex customer interactions in the public market with a series of statistical approximations. To evaluate the impact on model results, we ran the full and reduced models and produced trajectories of the following aggregate statistics over the course of the simulation:

- Average customer money
- Average customer drug inventory
- Average customer addiction level
- Average customer drug concentration

We then evaluated the goodness of fit between the full and reduced models on these statistics to decide whether the fit was adequate. Our model reduction procedure explored four versions of the reduced model: one which uses distributions for all five outcomes, one which uses a regression for the probability of obtaining heroin and distributions for everything else, one which uses a regression for the probability of being invited to make direct private deals and distributions for everything else and a final version which uses regressions for the probability of obtaining heroin and the probability of being invited to make direct private deals but distributions for everything else.

**Figure 8:**
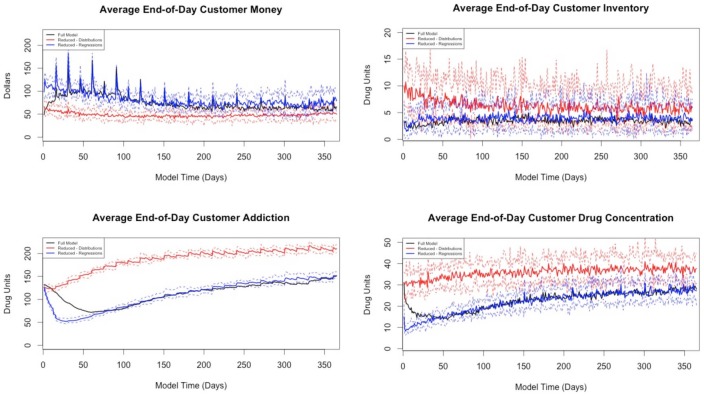
Comparison of full and reduced models. Dotted lines represent 95% intervals for reduced model output.

The average trajectories across 100 simulations of the full and reduced models are presented in Figure 8. In each plot we present the average output from the full model to the average output from two reduced models: one that used distributions to approximate all market decisions (the “distribution-based” reduced model), and another that used regressions for the probability of obtaining heroin and the probability of being invited to make direct private deals but distributions for everything else (the “regression-based” reduced model).

For all four statistics, the regression-based reduced model matches the full model more closely in both the magnitude and shape of the curves than the distribution-based reduced model. After day 180 of the simulation, which is the portion of the simulation we focused on because we believe the full model has moved beyond its transient state by that point, the regression-based reduced model matches the full model almost exactly.

**Figure 9:**
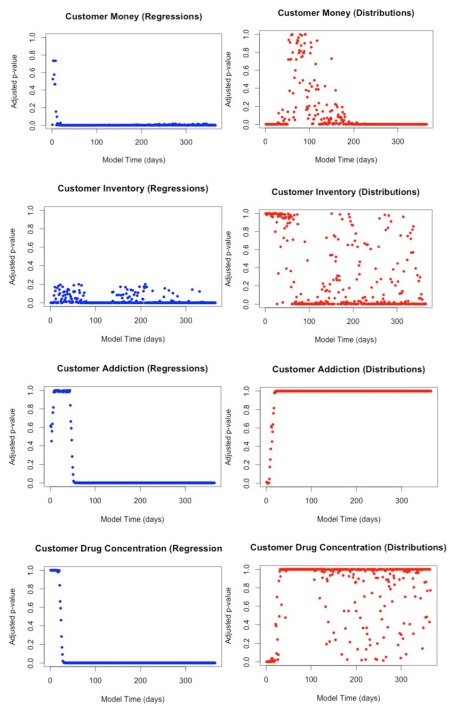
Adjusted *p*-values for each of the four quantities of interest comparing the reduced model based on distributions and the regression-based reduced model to the full model.

To compare the models more rigorously, we performed formal hypothesis tests comparing the means of the four statistics. We conducted these using point-wise tests at each simulated day and applied a multiple comparison correction procedure [13]. This correction procedure accounts for the fact that the type I error rate will increase as a result of performing 365 independent comparisons for each of our four output statistics for both versions of the reduced model. The resulting plots of the adjusted formal *p*-values comparing the full model to each version of the reduced model are presented in Figure 9. We would be satisfied if the distance between the trajectories is *smaller* than a certain value (e.g., 0.1 standard deviations) and thus calculate the *p*-value for that test.

This test is better suited for the purpose than a more traditional approach where failure to reject a null hypothesis of no difference is taken as the evidence of no difference [14]. This latter approach is not well suited for simulation studies because with a sufficient number of simulated trajectories one can make the *p*-value arbitrarily small. Furthermore, a null hypothesis that two values have a non-zero difference is principally flawed because the probability of two random numbers from continuous distributions being equivalent is zero. Thus, by increasing the number of simulations, any difference will become significant.

We thus employ a classic equivalence test [15] where we test that the difference is bigger than an a-priori defined value. In our case, the increase in the number of simulations will increase the precision and reduce the *p*-values if the effect size is smaller than the tested one (e.g., 0.1 standard deviations) and will never be reduced below some threshold value if the effect size is larger [16], [17]. This approach is also the basis for a classic power analysis, which is based on explicitly defining a minimally detectable effect size. If such minimally detectable difference is not pre-defined the test becomes powerless as the number of simulations increases.

The plots show that the regression-based reduced model has *p*-values at least as large as those for the distribution-based reduced model. There is strong evidence that the distribution-based reduced model's mean output for customer addiction and drug concentration are different from that of the full model, while the regression-based reduced model shows little to suggest that the means of these quantities are different from the full model.

The regression-based reduced model still shows some divergence from the full model, but the divergence occurs almost exclusively in the first 80 simulated days during the unstable transient period. For the distribution-based reduced model, the output improves in terms of comparison with the full model in the latter portion of the simulation, but, with the exception of customer inventory, the quantities do not match very well.

We also performed a two-sample Kolmogorov-Smirnov test for equivalence of the distributions of the summary statistics from the full and reduced models. In this test, the test statistic is
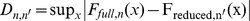
where n and n′ are the number of data points for each empirical distribution (in this case both values are 365) and x represents values in the data. The null hypothesis that the distributions are equivalent is rejected at a significance level of 0.05 if
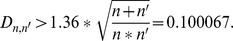



For all four summary statistics, the null hypothesis of equivalence of distributions is rejected for the reduced model using distributions and fails to be rejected for the reduced model with regressions. The results of the tests are presented in table 1 below.

**Table 1:** Results of two-sample Kolmogorov-Smirnov tests for both versions of the reduced model.

	Customer Money	Customer Drug Inventory	Customer Addiction Level	Customer Drug Concentration
Reduced model: distributions	0.23	0.34	0.67	0.55
Reduced model: regressions	0.076	0.051	0.093	0.087

In addition to the quality of the fit of the reduced model, we also saw a significant decrease in model runtime. The average runtime for a simulation of one year of market activity for the full model was 32.3 minutes, compared to an average of 18.3 minutes for the reduced model. This represents a 43.2% reduction in runtime.

## Addition of External Interventions

The model reduction procedure described so far was applied to a public heroin market that was free of external interventions. However, an important feature of the full model is its support of occasional police “busts”: increased police presence for a 24-hour period during which a number of street dealers are arrested and removed from the market (returning an average of 20 days later once they are released). We next sought to extend the reduced model to support police busts in the market.

We recognized that the five key customer market trip outcomes would likely vary depending on whether a bust is currently ongoing in the market. We first obtained new distributions or regression models for these outcomes exclusively during the bust periods. Then we modified the reduced model to use these new approximations while a bust was ongoing and to use the original approximations at all other times. For brevity, we have omitted the full details of the new distributions and regressions, but the following is a summary of how we approximated each of the five key market trip outcomes in the presence of busts:

- For the probability of obtaining heroin, because the distribution performed poorly in the non-bust scenario, we did not consider it here. We fit a new regression model using the data generated during busts. We replaced the original explanatory variable (amount of heroin sold by street dealers during the current day up to the time of purchase) with a new explanatory variable equal to the amount of heroin remaining in the street dealers' collective inventory at the time of purchase.
- For the probability of being arrested, we fit a distribution to the data generated during busts. We found the distribution to be sufficient when used in the reduced model.
- For the time spent in the market, we fit distributions to the data generated during busts. We found the distributions to be sufficient when used in the reduced model.
- For the probability of purchasing via a street broker, we fit a distribution to the data generated during busts, but we did not find the distribution to be sufficient when used in the reduced model. As a result, we fit a logistic regression model with a single explanatory variable equal to the ratio of the number of street brokers to the number of street dealers in the market at the time of purchase.
- For the probability of being invited to deal with a private dealer directly, because the distribution performed poorly in the non-bust scenario, we did not consider it here. We refit the regression model using the data generated during busts.

The new regression models for the probability of obtaining heroin and the probability of purchasing via a street broker included explanatory variables that we could not calculate using the original reduced model. Calculating these new explanatory variables required more detailed information about street dealers than our original reduced model retained. (Recall that we completely removed the street dealer agents from the original reduced model because we eliminated all their interactions from the model.) For this reason, we had to partially reintroduce street dealers to the reduced model. We did not implement them as full agents, though; instead we simply kept track of the number of street dealers currently in the market and the number currently arrested. From the full model, we estimated the rate of street dealer arrests while busts were not ongoing in the market, the rate of street dealer arrests while busts were ongoing in the market, and the rate of street dealers being released back to the market. We used these rates in the reduced model to continuously update the number of arrested street dealers.

**Figure 10:**
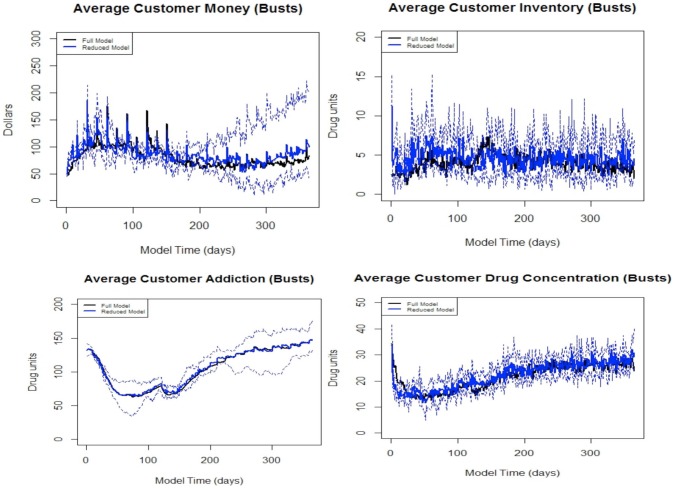
Comparison of full and reduced models including busts. Dotted lines represent 95% intervals for reduced model output.

**Figure 11:**
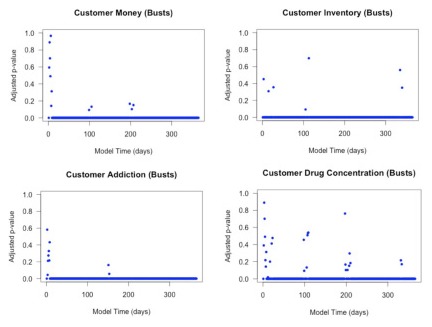
Adjusted *p*-values for each of the four quantities of interest comparing the reduced model to the full model including busts.

We conducted 100 simulations with police busts at days 120, 150, 180, and 210 in the full and reduced models. We examined the same set of aggregate statistics as in the earlier model reduction process, and we used the same hypothesis testing procedure to compare the two models. The results from the reduced model match the full model remarkably well, and the hypothesis tests show little evidence to suggest that the means of the reduced and full models differ for any of the summary statistics after incorporating busts into the model. We present the comparisons in Figures 10 and 11.

## Discussion

We created a reduced version of the full IDMS agent-based model that performs equivalently to the full model. We identified key aggregate model statistics and a semiformal way to assess the equivalence of the models. Although the reduced model is still an agent-based model and has a discrete timestep (1 minute), it has a much simpler structure and the runtime is reduced.

We focused on simplifying the model from the perspective of the customer agent. However, it is possible to develop a reduced model from the perspective of a different type of agent, such as the police officer agent. It is also possible to further reduce the model and explore the limit of the emulation (i.e., finding the simplest model that would generate trajectories similar to the ones produced by the full model). Although finding the minimal emulator is feasible and theoretically interesting, the more the model is simplified the less feasible it becomes to examine the effect of specific interventions. Such exercises, however, are beyond the scope of the current paper.

The procedure of model comparison has utility beyond the development of the reduced model. Comparative analysis of different drug markets is an issue facing ethnographic researchers [18]. The original model addressed this issue by creating a framework for comparing different drug markets, and the techniques described here advance the ability to perform this comparative analysis. Such analysis also allows one to validate the model when only aggregate data are available. One still must keep in mind that a number of very different reduced models can produce the same aggregate behavior and the way to select between alternative models is to examine scenarios where the models diverge.

In the process of creating the reduced model, we encountered a number of challenges and learned a few lessons. When attempting to reduce a complex agent-based model, the statistics used for model comparison should be determined a priori and are likely to be aggregated across individuals, such as average customer money in our case. The challenge is to identify a manageable set of individual statistics which capture the dynamics of the original model and, when replaced with the emulated values, would produce adequate model outcomes.

While the space of behavior of this model is quite large, we focused this analysis on a reduced set of the model space, namely behavior and outcomes directly related to customer agents. This restriction made our procedure more straightforward in that it limited the set of features of the heroin market (and the corresponding features of the full model) that needed to be considered and analyzed.

The issue of the optimal timestep in simulations has been extensively discussed in mathematical, computational, and statistical literature [19]–[21]. As the timestep becomes larger, the accuracy of the simulation model falls. At the same time, when the timestep becomes unnecessarily small, small inaccuracies such as rounding errors accumulate and lead to distortions. In our study we observed similar phenomena.

The remaining gap in theoretical development is the choice of analysis variables and models to represent these variables and distributions. We explored numerous relationships between individual characteristics by using spline fitting and trellis graphics to visualize nonlinearities. As a result, we learned which relationships are important to track and which are causal to the model's behavior. For example, the main factor that affected the probability of successfully obtaining the drug was not the number of other customers in the market, as we first assumed, but instead was the amount of available drug.

Although the reduced model produces behavior similar to the full model for existing scenarios, introducing a new scenario is likely to require development of a new reduced model that specifically accounts for the features of the new scenario. We encountered this issue when we introduced an external intervention in the form of police busts. The robustness of the model to the initial conditions has been described elsewhere [3] (it converges to a stable dynamic regime). The presented exercise is an example of showing the robustness of the reduced model to the external shocks. The reduced model was developed when the amount of police did not substantially change. The introduction of the “busts” means that during a short period of time a large portion of street dealers gets arrested which introduces shocks to the system. Both the full and the reduced model show that they respond similarly to these shocks, which is an encouraging observation.

Another complication when reducing an agent-based model is the adaptive nature of the agents. In the full model, customers have a learning process by which they gain information about street sellers (their locations and the success or failure of a transaction with them) and private dealers. As customers gain this information, they adapt to maximize their prospects of obtaining the drug. Although this is in line with current research [11], this adaptation should be accounted for by creating dynamically changing distributions. In our regression-based reduced model, the learning process is captured only through customers being put in contact with private dealers based on their number of successful street broker transactions. We hypothesize that this alteration of the learning process contributes to the divergence in the trajectories of the models during the transient period of the first 180 or so days of the simulation.

Models based on ethnographic research have the limitation that only the features included in the research can be built into the model [22]. As a result, the reduced model is constrained by the features included in the full model. If the full model, however, contains a sufficient amount of detail, the reduction process allows us to identify which of the factors are most influential on the model outcomes. These analyses would inform social sciences about the relationships in the real-life processes which were represented by the model.

In this paper we present a reduced model based on the original IDMS model. As the IDMS model is developed further, we plan to explore applying this model reduction technique to the revised versions of the model. A potential benefit of the reduced model is that is allows a flexible range of experimentation in simulating market activity. One of the future plans for the IDMS model is to perform experimentation through the introduction of social networks in the reduced model to describe cooperation among customers in obtaining heroin. Social networks will provide additional features to be considered during the model reduction process. For example, the probability of joining a new network may be an important individual outcome that can be replaced by a distribution or regression model, network size may be a useful covariate for the regression models, and synchronization of drug habits within customer cliques may be a new model outcome to use when comparing the full and reduced models.

Employing the reduced model over the full model is beneficial a data collection standpoint. Having established a simplified set of summary statistics and output measures of interest, this limits the information one would have to obtain to further study the behavior of this market. This is an important feature of reduction of ABMs in general, in that it constrains the information needed for further study of a system to a specific set of well-defined quantities. This also provides insight into how one could validate such a reduced model given our focus on explanatory value and our well-defined set of behaviors of interest. Our reduced model is also advantageous from an educational standpoint since it allows for a concise description of the heroin market. Reduced models such as that which we have presented allow an straightforward explanation of the function of a system and an intuitive assessment of the specific agent behaviors that affect the significant outcomes of the model.
